# Energy metabolism and partition of lactating Zebu and crossbred Zebu cows in different planes of nutrition

**DOI:** 10.1371/journal.pone.0202088

**Published:** 2018-08-17

**Authors:** Pedro Henrique de Araujo Carvalho, Ana Luiza da Costa Cruz Borges, Ricardo Reis e Silva, Helena Ferreira Lage, Paolo Antônio Dutra Vivenza, José Reinaldo Mendes Ruas, Elias Jorge Facury Filho, Rodrigo Liberio Araújo Palhano, Lúcio Carlos Gonçalves, Iran Borges, Eloísa de Oliveira Simões Saliba, Diogo Gonzaga Jayme, Antônio Último de Carvalho

**Affiliations:** 1 Federal University of Minas Gerais, Veterinary School, Belo Horizonte, Minas Gerais, Brazil; 2 Department of Animal Science, Unimontes, Janaúba, Minas Gerais, Brazil; University of Illinois, UNITED STATES

## Abstract

The aim of this study was to determine the energy metabolism and partition of lactating Gyr and F1 Holstein x Gyr (F1 HxG) cows in different planes of nutrition. Six F1 HxG and six Gyr cows with 130 days in milking (DIM) fed corn silage and concentrate were evaluated. The experiment consisted of four periods with different levels of feeding: 1st *ad libitum* dry matter intake (DMI) and the others with 5, 10 and 20% restricted DMI, related to the first one. An apparent digestibility assay was performed before measurements in the respiration chamber. Total feces were collected for three days. The cows were confined for 24h in the chamber in each period to determine methane and heat production (HP). F1 HxG had higher gross energy intake (GEI), metabolisable energy intake (MEI) and digestible energy intake (DEI). GE lost in feces was higher in F1 HxG (23.7% GEI) than in Gyr (20.5%) cows. Energy lost as methane and urine was similar between the groups. The metabolisability (q) was 0.67, and the efficiency of converting ME to NE (k) was 0.56. There was no difference in the energy requirements for maintenance between breeds (426.6 MJ/kg BW^0,75^ average value). The energy requirements for lactation were higher in F1 HxG animals due to the higher volume of milk produced, since there was no difference in energy requirements for production of one kg of milk.

## Introduction

Zebus and their crossbreed with *Bos taurus* animals represent more than 70% of Brazil’s dairy cows. The main breed used to cross with Holstein (H) animals is Gyr (G). F1 HxG cows have benefits from heterosis, while combining the main features of each breed. They have good milk production and are adapted to tropical conditions.

Zebus and their crossbreeds are the basis of the Brazilian dairy herd, and milk production in Brazil has been increasing. has been showing a significant increase in milk production. They are very rustic animals and their male calves are used for fattening. Due to this dual purpose characteristic, when supplemented for increased milk production, they often gain a lot of weight in the final phase of lactation, reaching high body condition scores, which can lead to problems in subsequent lactation. Thus, food restriction in the middle and final lactation can be a viable alternative to avoid excessive in these animals. However, there are no data regarding metabolism and energy partitioning of these animals that allow to establish an adequate nutritional management with a viable cost and without compromising the productivity of the animals.

The aim of this study was to determine the energy requirements for maintenance and lactation, and study the energy partition between maintenance lactation of Gyr and F1 HxG from 130 DIM to the end of lactation at different levels of feeding.

## Material and methods

### Location of the study

The procedures adopted in this research were approved by the Ethics Committee and Animal Experimentation of the Federal University of Minas Gerais (Permit number 184–2015). The experiment was conducted at the Metabolism and Calorimetry Laboratory of the Animal Science Department at the Veterinary School of the Federal University of Minas Gerais, located in Belo Horizonte, Brazil.

### Cattle breed and housing facilities

Twelve cows with 130 days in milking (DIM), six Gyr and six F1 Holstein x Gyr (F1 HxG) were used. F1 HxG cows had 510.29kg medium live weight (LW) and produced 4833.9 kg milk throughout lactation. Gyr cows had 482.29kg LW and produced 2603.5 kg. The animals were first-calving animals, already in adult age and weight, that is, no longer presenting nutritional requirements for growth. Animals were housed in a tie-stall system.

### Diets and experimental management

The diet (Table 1) was corn silage and the concentrate diet a 60:40 corn silage:concentrate ratio to supply the nutritional requirements for maintenance, lactation and average daily gain of 200 g/day according to [1]. The concentrate was composed of ground corn, soybean meal and mineral supplement. Each experimental period lasted 28 days.

**Table 1:** Chemical composition of corn silage and concentrates offered to Gyr and F1 Holstein x Gyr cows, expressed as a percentage of dry matter.

Nutrient	Corn silage	Concentrate
DM*	28.83*	88.0*
OM	91.56	96.01
CP	8.10	23.64
EE	3.88	3.48
NDFap	47.78	21.54
ADF	26.26	4.59

DM—Dry matter;

* Expressed as a natural matter; OM—organic matter; CP—crude protein; EE—ether extract; NDFcp—neutral detergent fibre correct to ashes and protein; ADF—acid detergent neutral

The experiment was composed of four periods with different levels of feeding: 1^st^- *ad libitum* dry matter intake (DMI), 2^nd^—restriction of 5%, 3rd—restriction of 10% and 4^th^- restriction of 20% DMI, related to the 1^st^ period. The animals were fed twice a day immediately after milking. During the restricted periods, crude protein (CP) and minerals were corrected according [1].

### Milking and milk collection

Milking was performed twice a day, at 6 am and 3 pm. Daily milk production was measured and milk samples were collected before the respiration chamber evaluation. Milk composition analysis (fat, protein and lactose) were performed in the Quality Analysis Laboratory of the UFMG Milk laboratory (LabUFMG)

### Apparent digestibility assay and urine collection

During each period, after 21 adaptation days, an apparent digestibility assay was performed. Total feces were collected for three days. The urinary volume was estimated by spot collection according to [2].

### Calorimetry and energy balance calculation

The cows were confined in the respirometry chamber after the digestibility assay. The procedures and system specification have been described by [3]. Heat production measurement was carried out during 24h with animals fed the same diet outside the chamber and after 48h solid food fasting. Oxygen consumption (O_2_) and carbon dioxide (CO_2_) and methane (CH_4_) produced were registered. Gas and heat Energy losses were measured. Volumes (L/day) of O_2_ consumed, CO_2_ and CH_4_ produced in 24 hours, and urinary nitrogen excreted (Nu, g/day), were used to estimate heat production (HP), according to [4]: HP (KJ) = 16.18 O_2_ + 5.02 CO_2_−2.17 CH_4_−5.99 Nur. Metabolisable energy (ME) was determined by subtracting the gross energy (GE) of feces, urine (adiabatic calorimeter PAAR-1281 model) and methane from the gross energy intake (GEI). GE loss as methane was calculated by assuming the loss of the 39.5 KJ/L CH_4_ produced. Digestible energy (DE) and ME in the diet, expressed as MJ/kg DM, were obtained during metabolism assay. Heat increment (HI) was determined by subtracting fasting heat production (FHP) of heat output of the fed animal. Basal metabolism or nutritional requirement for maintenance was determined in respirometric chamber after 48 h of fasting from solid foods, after the end of the four periods. Energy requirements for milk production were determined by combustion heat obtained from each component of milk, fat, protein and lactose [1], multiplied by milk production. Energy available for body weight gain or loss and energy balance was calculated by subtracting the values of heat produced by fed animal and net energy from the milk.

### Statistical analysis

The experimental design was completely random, each animal was an experimental plot. Two treatments with six replicates each were used. Four subplots were used, each subplot being a nutritional plain. Average values were compared by Tukey test at 5% probability (P <0.05).

## Results and discussions

Table 2 expressed the energy balance at the different nutritional plans.

**Table 2:** Daily Energy partition of Gyr and F1 Holstein x Gyr cows in different nutritional plans.

	GYR				F1 HxG				SEM*	SEM**
	*AL*	5%	10%	20%	*AL*	5%	10%	20%		
**GEI MJ**	201.8^Ba^	176.72^Bb^	170.8^Bb^	165.8^Bb^	279.7^Aa^	241.9^Aa^	229.4^Aab^	220.6^Bb^	1.4	0.8
GEf MJ	40.2^B^	32.6^B^	37.7^B^	36.8^B^	69.9^A^	58.6^A^	50.2^A^	51.9^A^	0.6	0.5
GEf /GEI %	19.9^B^	18.4^B^	21.7^B^	21.9^B^	25.1^A^	24.3^A^	21.9^A^	23.7^A^	0.9	1.6
**DEI MJ**	161.6^B^	144.0^B^	133.5^B^	128.9^B^	209.7^A^	182.9^A^	179.2^A^	168.3^A^	1.1	0.8
CH_4_ / day L	299.5^B^	278.6^B^	280.5^B^	289.1^B^	371.6^A^	346.9^A^	360.8^A^	364.4^A^	1.3	1.1
CH_4_/Kg DM	5.8^B^	6.3^B^	6.5^B^	6.5^B^	5.2^A^	5.7^A^	6.2^A^	6.9^A^	1.1	2.0
GEu MJ	7.9	7.1	7.5	7.5	8.8	10.0	8.4	9.6	0,6	0.9
GEu/GEI %	4.0	4.2	4.4	4.6	3.2^b^	4.1^a^	3.7^a^	4.4^a^	0.1	0.1
**MEI MJ**	141.9^Ba^	125.2^Bab^	114.7^Bb^	109.7^Bb^	185.9^Aa^	160.8^Ab^	156.2^Ab^	144.0^Ab^	0.2	0.2
HI MJ	61.9	59.4	53.2	39.8	81.2	61.9	61.9	63.6	0.6	0.9
HI / GEI %	30.8	33.9	31.9	28.8	28.7	25.7	27.1	24.1	1.1	1.8
HI/Kg DM	6.3	5.9	6.7	5.0	5.4	4.6	4.6	5.4	0.1	0.4
**NE**_**L**_ **MJ**	29.7^B^	29.7^B^	33.5^B^	30.6^B^	57.3^A^	56.5^A^	58.6^A^	54.0^A^	0.7	0.1
Milk Kg/day	9.0 ^Ba^	8.7 ^Ba^	9.6 ^Ba^	8.2 ^Bb^	17.6 ^Aab^	17.3 ^Ab^	18.2 ^Aa^	16.1 ^Ac^	0.9	0.3
NE_L_ MJ/L	3.5	3.8	3.6	3.4	3.3	3.3	3.3	3.4	0.1	0.1
NE_L_ /GEI	14.4^Bb^	16.4^Ba^	19.5^Ba^	18.4^Ba^	20.6^Ab^	23.4^Aab^	25.6^Aab^	24.5^Aa^	1.4	0.8
**NE**_**m**_ **MJ**	41.7	41.8	41.5	40.9	46.5	47.8	47.7	47.6	0.4	0.1
**NE**_**m**_ **kJ/BW**^**0,75**^	408.3	408.3	408.3	408.3	445.7	445.7	445.7	445.7	3.3	0.4
NE_m_/GEI	16.7	19.8	20.9	21.6	20.7	23.8	24.6	24.9	1.5	0.4
**EB MJ**	3.5	-5.4	-13.8	-1.7	0.1^b^	-7.1^b^	-12.1^b^	-21.3^a^	1.1	1.6

Average followed by different capital letters on the same line differ among themselves by the Tukey test at 5% probability in the comparison between the genetic groups; Average followed by different lowercase letters on the same line differ by Tukey test at 5% probability in the comparison, in the same genetic group, in the different alimentary planes. AL–*Ad libitum*; GEI—gross energy intake; GEf—fecal gross energy; DEI—digestible energy intake; CH4 —methane; GEu—urine gross energy; MEI—metabolisable energy intake; HI—heat increment; NE_L_—lactation net energy; NE_M_—maintenance net energy; EB—energy balance.

*—SEM genetic group;

**—SEM nutritional plan

### Ad libitum plan

GEI was higher in F1 HxG (279.67 MJ/day) compared to the Gyr group (201.8 MJ/day). [5], evaluating the size and weight of internal organs of *Bos indicus*, found that these animals had smaller digestive tract than *Bos taurus*. This justifies the largest DMI by animals F1 HxG (14.8 kg DM/day) compared to Gyr (9.9 kg DM/day), associated with higher milk production by F1 HxG compared to Gyr. [6], in a meta-analysis on productive performance of lactating crossbred cows, found 15.5 kg DMI, a value close to that presented by F1 HxG. The highest GEI can be justified only by the difference in DMI, since the diet between the groups had the same chemical composition, which would be a possible source of variation in the dietary energy content. [7] found 276.33 MJ GEI/day in crossbred taurine lactating animals.

Feces GE was higher in F1 HxG, (69.92 MJ/day) compared to Gyr (40.19 MJ/day). This result was expected due to higher DMI in animals F1 HxG. However, the wasted energy in feces compared to GEI was higher in animals F1 HxG (25.1%) compared to Gyr (19.9%). According to [8], higher levels of DMI, and therefore GEI, increase the digesta passage rate, reducing food utilization and increasing the energy loss in feces. This better dietary utilization in the Gyr animals, which presented lower intake, was observed in the apparent digestibility of the diet (P <0.05). The apparent digestibility of the diet was 0.756 g / kg in the Gyr and 0.712 animals in the F1 HxG animals. [7] did not find breed effects between crossbred and purebred cattle, with average values of 22.4% of GEI lost in feces.

Digestible energy intake (DEI) was higher in F1 HxG animals compared to Gyr (209.76 and 161.61 MJ/day, respectively). This finding was expected, since GEI was higher in F1 HxG.

Daily methane production was higher in F1 HxG (371.6 L) animals compared to Gyr (299.5 L). Comparing methane production and GEI there was no difference between genetic groups (P <0.05), with an average value of 5.5% GEI as methane. [9] studied same breed heifers and found average values of 5.9% GEI as methane. These results are close to the proposed limit of 5.5 to 6.5% GE lost as methane in lactating animals [10].

Energy lost as urine was similar between genetic groups, with an average value of 3.7% GEI. [11] and [12] found values of 3.5 to 6% EB lost as urine in lactating and growth animals.

Since GEI and DEI were higher in F1 HxG, there is no difference on the percentage loss as methane and urine, MEI was higher in F1 HxG animals (185.9 MJ/day) compared to Gyr (141.9 MJ/day). [13] found values close to those of Gyr cows, 149.89 MJ/day.

Heat increment (HI) was similar between the genetic groups, with an average value of 77.87 MJ/day. HI and GEI ratio was similar too, with an average value of 29.7% of GEI lost as HI. [14] proposed a range from 20 to 42% of GEI lost as HI; for lactating animals, these values would be close to 30%. Gyr cows showed higher HI/kg DMI, but within the range proposed by [14]. This could possibly be explained due to the double fitness of these animals which have the ability for milk production and weight gain, and their conversion efficiency of ME to NE for weight gain is lower.

Net energy requirement for milk production (NE_L_/kg milk) was similar between the genetic groups, 3.43 MJ/kg milk. This value is close to that of the [1] for the production of same composition milk. [15] propose that net energy for animal products is equivalent to the combustion heat of milk components, fat, protein and lactose. Due to the higher milk production presented by F1 HxG, the NE_L_ per day was higher in these animals (57.36 MJ/day), in relation to the Gyr (29.73 MJ/day).

There was no difference between the nutritional maintenance requirements between breeds, with an average value of 426.6 MJ/kg BW^0.75^. According to [4], the fasting heat production (FHP) is equivalent to the energy maintenance requirements. [16], using the comparative slaughter technique, determined FHP as 322.38 MJ/kg BW^0,75^. The [17] table uses 322.38 MJ/kg BW^0,75^, and [1] adopted 334.9 MJ/kg BW^0,75^, considering corrections for differences between empty body weight of the animals and activity fulfilled. International committees such as the [1], [18] and [19], suggest an increase from 10 to 20% in the nutritional requirements due to changes in lactation, such as the increase of blood circulation to supply more glucose to the mammary gland, causing an increase in activities of organs as heart and lungs. There is also an increase of liver activity for carrying out processes such as gluconeogenesis. These organs, although representing approximately 5–6% of the live weight of the animal, require much of its energy demand [20].

[21] compared energy requirement for maintenance in Holstein and Jersey animals and found no difference between these breeds. However, both breeds evaluated by these authors were from bovine origin and had high milk production. High milk production capacity may be able to dilute the maintenance requirements in relation to the animals’ total requirement. In Zebus and crossbred cows, which have lower milk yield potential, energy requirements for maintenance can represent a larger share of the total net energy requirements and require therefore further study. These animals are the basis of the Brazilian dairy herd, and the genetic improvement has been showing a significant increase in milk production. They are very rustic animals and their male calves are used for fattening. Due to this dual fitness characteristic, when supplemented for increased milk production, they often gain a lot of weight in the final lactation phase, reaching high body condition scores, which can lead to problems in subsequent lactation. Thus, food restriction in the middle and final lactation can be a viable alternative to avoid excessive tissue deposition in this period, without compromising the milk production in these animals.

The energy balance was similar between the breeds, with an average value of 1.8 MJ/day. According to [1], for animals with similar body condition score, at the same lactation stage, the NE value available for weight gain would be enough to promote weight change of 0.072 kg per day, that is, these animals had an energy balance close to zero. Animals in the middle third of lactation have passed the peak of production and have re-established the DMI. An adequate DMI can meet the nutritional requirements, leaving a negative energy balance. Thus, weight changes tend to be small at this stage, as happened in this experiment [22]. [6] evaluated the lactation and intake curve throughout the lactation of three different crosses between zebu and Holstein cows, one of the groups was HxG. The authors observed that crossbred animals showed higher consumption in the sixth week of lactation and presented a lower negative energy balance period, presented by Holstein animals.

### Feed restrictions

The feed restrictions imposed were 5, 10 and 20% in relation to ad libitum plan. However, for behavioral issues, some animals did not consume all diet provided during dietary restrictions. For this reason, the real nutritional constraints observed differed from the one initially proposed. The values presented by the animals Gyr of 12.4; 15.4 and 17.8% of the ad libitum plan. In the F1 HxG animals the values were 13.5; 18.0 and 21.1% of the ad libitum plan.

**Fig 1:**
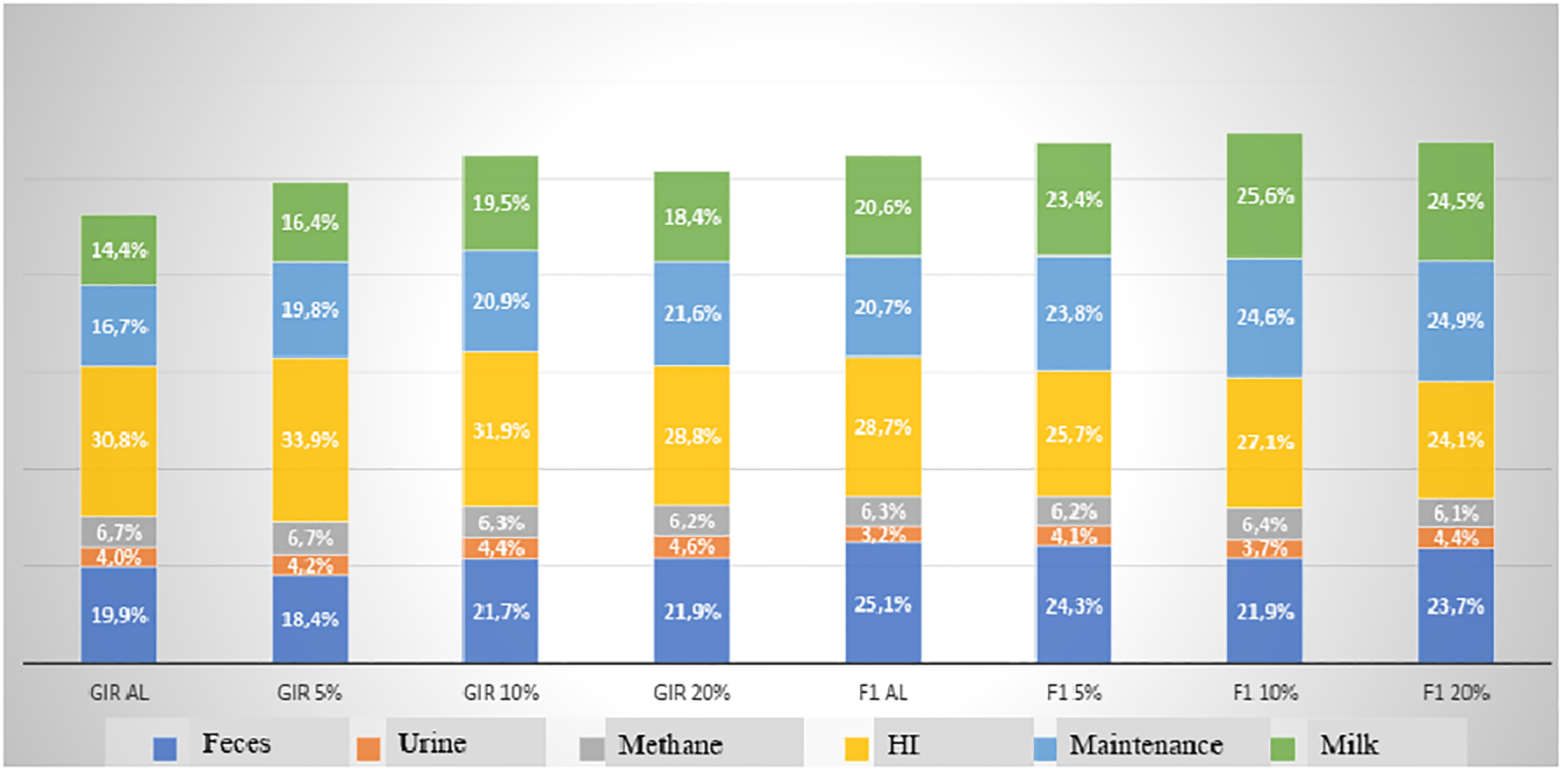
Daily Energy partition of Gyr and F1 Holstein x Gyr cows in different nutritional plans. (HI:heat increment).

The effect of feed restrictions inside the genetic groups was not evaluated because was the treatment, thus should not technically be statistically analyzed. The restrictions were intentionally mild in order to promote changes in the digestive process resulting in a same milk production without effect in body condition. The percentage of GE lost as feces was higher in F1 HxG (23.3%) compared to Gyr (20.7%), as occurred in the 1^st^ period (Fig 1). [12], evaluating the scheduled dietary restriction in finishing steers, did not observe differences in the percentage of energy lost in the feces between the different restrictions, 10 and 20%, being these values lower than that presented in the *ad libitum* phase. MEI was similar among 2^nd^, 3^rd^ and 4^th^, being higher in F1 HxG in relation to Gyr.

Daily CH_4_ production did not differ between periods in both genetic groups. F1 HxG cows produced more CH_4_ than Gyr, 357.4 and 282.7 L/day, respectively. However, the percentage of GE lost as methane showed no difference between the genetic groups, averaging 6.2% (P <0.05). The percentage of GEI lost as urine in food restrictions was higher, 4.1% compared to that shown in the *ad libitum* phase in F1 HxG animals, Gyr cows showed no difference between periods. [12], evaluating dry matter restriction levels like those of the present study (10 to 20%) reported increased energy lost as urine depending on the dietary restriction.

MEI showed no difference between the restrictions in animals F1 HxG, averaging 153.6 MJ/day, being lower than in the *ad libitum* period. In Gyr animals, in the 10% and 20% restrictions, it was lower than the other periods and with equal values among them: 112.2 MJ/day. In the 5% restriction, a 125.2 MJ/day MEI was intermediate without differences with the other restrictions and the ad libitum plane. At all stages, the MEI values presented by the F1 HxG animals were higher than those presented by Gyr.

HI showed no difference between restrictions periods and between genetic groups, averaging 56.6 MJ/day. Just as there was no difference in HI/kg DM between the genetic groups or periods, averaging 5.4 MJ/kg DMI. [13] reported the superiority of HI in dairy cows with feed restriction, with a 3% increase in the HI related to GEI. According to [14], HI in lactating animals has values near to 30%, but this value can reach up to 42% in weight gain per animal.

Energy directed to milk production did not differ between the periods. In all periods, energy directed to milk production was higher in F1 HxG than in Gyr, due to higher milk production presented by the F1 HxG animals. However, the relationship between GEI and energy directed to milk production was higher in the 20% restriction and in the *ad libitum* diet. 5 and 10% restricted diets showed intermediate values between the two phases. These diets demonstrate greater efficiency for milk production in food restrictions, since a higher percentage of energy intake was directed to milk production. All animals were evaluated weekly, and there was no change in body score condition greater than 0.5.

The energy deficit due to the feed restriction was greater in the 20% restriction in animals F1HxG (-21.3 MJ/day) in comparison with other phases, which were averaged -6.4 MJ/day. In Gyr animals there was no difference between the periods, with an average energy deficiency of -6.9 MJ/day.

Elm was higher in F1 HxG (47.7 MJ/day) compared to Gyr (41.4 MJ/day). This difference was due to higher body weight presented by animals F1 HxG, since there was no difference in fasting heat production among the genetic groups in MJ/BW^0,75^.

The relationship between ME and GE, "q" or metabolisability coefficient, was similar between the genetic groups or between periods, with an average of 0.67. this means that 67% of the GEI was saved as ME. [7] found no difference for metabolisability between taurine and crossbreed animals fed the same diet with different levels of concentrate, with average value of "q" of 67.2. [23], working with F1 HxG bulls fed corn silage and concentrate in different nutritional planes, found different values of metabolisability as a function of the nutrition plane. In animals fed near maintenance, this coefficient was 0.65, and planes with *ad libitum* feeding had 0,61 metabolizability. Efficiency to convert ME in NE, “k” value, has been reported by the [1] values close to 0.64. However, these values are determined in taurine animals and in different conditions presented in Brazil. [24], working with Zebu bulls found lower values of 0.56. It is important to note the lack of zebu animal data assessed in lactating respirometric chambers as a limiting factor for further discussion regarding the efficiency of energy use by these animals.

## Conclusion

F1 HxG animals showed higher intake of gross energy, digestible energy and metabolisable energy in relation to Gyr. There was no difference in the percentage of gross energy lost as urine between the genetic groups and dietary restrictions, and there was no difference in the efficiency of energy use expressed by the coefficients "q" and "k". The energy directed to milk production was higher in F1 Holstein x Gyr animals, the same happened with the daily production of methane. Feed restriction until 10% of DMI does not compromise the milk production or body condition in zebus or F1 HxG.

## Supporting information

S1 Table This is raw data set used to reach the conclusions drawn in a manuscript. (XLSX)
